# Robustness of testing procedures for confirmatory subpopulation analyses based on a continuous biomarker

**DOI:** 10.1177/0962280218777538

**Published:** 2018-06-11

**Authors:** Alexandra Christine Graf, Gernot Wassmer, Tim Friede, Roland Gerard Gera, Martin Posch

**Affiliations:** 1 Center for Medical Statistics, Informatics, and Intelligent Systems, Medical University of Vienna, Austria; 2 Department of Medical Statistics, University Medical Center Goettingen, Germany

**Keywords:** Subpopulation analysis, group sequential design, multiple testing, biomarker, prognostic effect

## Abstract

With the advent of personalized medicine, clinical trials studying treatment effects in subpopulations are receiving increasing attention. The objectives of such studies are, besides demonstrating a treatment effect in the overall population, to identify subpopulations, based on biomarkers, where the treatment has a beneficial effect. Continuous biomarkers are often dichotomized using a threshold to define two subpopulations with low and high biomarker levels. If there is insufficient information on the dependence structure of the outcome on the biomarker, several thresholds may be investigated. The nested structure of such subpopulations is similar to the structure in group sequential trials. Therefore, it has been proposed to use the corresponding critical boundaries to test such nested subpopulations. We show that for biomarkers with a prognostic effect that is not adjusted for in the statistical model, the variability of the outcome may vary across subpopulations which may lead to an inflation of the family-wise type 1 error rate. Using simulations we quantify the potential inflation of testing procedures based on group sequential designs. Furthermore, alternative hypotheses tests that control the family-wise type 1 error rate under minimal assumptions are proposed. The methodological approaches are illustrated by a trial in depression.

## 1 Introduction

With the advent of personalized medicines, clinical trials studying treatment effects in subpopulations have gained more and more attention. The objective of such studies is to identify subpopulations based on biomarkers, where the treatment has a positive effect. Here the term biomarker is used in a very general sense as a synonym for a baseline patient characteristic, like demographic, clinical or genetic variables or a combination of these. They are measured prior to treatment and therefore cannot be affected by the outcome. For example, there is an extensive discussion in the literature whether biomarkers can be used to predict the treatment effect of medicines in patients with depression.^1,2^ Although a number of treatment options for such patients are available, no single treatment is universally effective. Biomarkers can be prognostic or predictive, where prognostic biomarkers predict the outcome in a natural cohort, and predictive biomarkers, in contrast, predict the treatment effect of an experimental treatment in comparison to a control group.^3^ Note that biomarkers may be both prognostic and predictive.

A wide range of methods for the identification and confirmation of targeted subpopulations in clinical trials has been proposed.^4^ Several authors focused on settings, where subpopulations are defined by a continuous biomarker which is dichotomized to define biomarker-low and biomarker-high subpopulations. The subpopulation with an expected beneficial treatment effect is called the biomarker positive subpopulation and the complementary subpopulation is called the biomarker negative subpopulation. Then, hypotheses tests to test for treatment effects in the subpopulation of biomarker positive patients and the full population are performed. Because several hypotheses are investigated, an appropriate multiple testing procedure has to be applied to control the family-wise type 1 error rate (FWER).^5–7^

An important problem is the choice of the threshold. To obtain a conservative hypothesis testing procedure to test for treatment effects in subpopulations, the considered threshold needs to be defined a priori, either based on an independent data set or theoretical considerations. If there is uncertainty regarding the choice of the threshold, more than one threshold may be investigated. The nested structure of subpopulations defined by different thresholds for a continuous biomarker is similar to the structure of analysis populations in group sequential trials. Hence, it has been proposed to use critical boundaries of group sequential designs^8^ to test nested subpopulations.^6,9^ However, the validity of these designs depends on the assumption that the variance of the outcomes does not vary across subgroups.

In this paper, we show that great care has to be taken when applying group sequential boundaries to test hypotheses for multiple nested subpopulations as proposed in the literature.^9^ We show that for biomarkers with a prognostic effect that is not adjusted for in the statistical model, the variability of the outcome may vary across subpopulations. As this may have an impact on the correlation of the test statistics, the use of group sequential boundaries may not guarantee control of the FWER. Using simulations, we quantify the potential inflation of the FWER of testing procedures based on such group sequential designs. To obtain test procedures that control the FWER, we show how inverse normal combination tests^10^ and sequential regression tests^8^ can be applied to this testing problem. Furthermore, we consider a test accounting for the different variances across subgroups^6^ and propose a modification of this test that accounts for the respective degrees of freedoms of the test statistics using the quantile substitution method. We show that the latter procedure controls the FWER under minimal assumptions and compare its power under a range of scenarios to alternative approaches. In addition, we generalize the multiple *t*-test to general subgroup tests for non-nested subgroups. To illustrate the procedures, we give a clinical trial example in depression.

## 2 Statistical model and testing problem

Consider a randomized parallel group clinical trial designed to evaluate a novel treatment compared to a control with a per group sample size of *n*. For simplicity, equally sized groups are assumed. For each subject i=1,…,2n, a continuous biomarker *X_i_* is observed and, due to the sampling of patients, we assume that the *X_i_* are independent draws from some distribution. The biomarker *X_i_* may be prognostic for the outcome *Y_i_* and/or predictive for the treatment effect such that

(1) $$Y_{i}=\beta_{0}+\beta_{1}U_{i}+\beta_{2}f_{1}(X_{i})+\beta_{3}U_{i}f_{2}(X_{i})+\varepsilon_{i},i=1,\ldots ,2n$$

where *U_i_* = 1 (0) if a subject is allocated to the treatment (control) group and the treatment assignments *U_i_* are assumed to be statistically independent of *X_i_* and *ε_i_*. $f_{1}(X)$ and $f_{2}(X)$ are functions characterizing the prognostic and predictive effect of the biomarker. Without loss of generality, it is assumed that the biomarker variable *X_i_* takes values between 0 and 1. The error terms *ε_i_* are assumed to be normally distributed with mean 0 and variance $\sigma^{2}$. Let $y_{i},u_{i},x_{i}$ denote the observed values of the outcome, treatment assignment, and biomarker of subject i=1,…,2n.

Consider an analysis strategy with the goal to identify a (sub)population, defined by a dichotomization of the biomarker *X_i_*, where the treatment has a positive effect. To this end, we consider nested subpopulations $S_{+}(q_{k})$ (which we call biomarker positive populations), based on increasing pre-specified thresholds $q_{k},k=1,\ldots ,K$ given by

$$S_{+}(q_{k})=\{i:x_{i}\leq q_{k}\}$$

Thus, here the biomarker positive subpopulations (for which a positive treatment effect is expected) consist of all patients with biomarker values below the threshold *q_k_* (later we will also discuss the case of more general types of biomarker positive subgroups).

Separate hypotheses tests in the biomarker positive subpopulations could be considered, e.g., if there exists prior information that the treatment effect in a biomarker positive population may be larger than in the corresponding biomarker negative population; however, insufficient information on the dependence structure of the outcome on the biomarker is available and therefore several thresholds are investigated. To confirm a positive treatment effect in the considered subpopulations, we compare the mean responses of the treatment and control group of each subpopulation $S_{+}(q_{k})$. Let $\mu_{t}(q_{k})=E(Y|U=1,X\leq q_{k}),\mu_{c}(q_{k})=E(Y|U=0,X\leq q_{k})$ denote the means and $\sigma_{t}^{2}(q_{k}),\sigma_{c}^{2}(q_{k})$ the respective variances of the outcome *Y* for the subpopulation $S_{+}(q_{k})$. We then test the *K* null hypotheses

(2) $$H_{0k}:\delta (q_{k})\leq 0\text{ against }H_{1k}:\delta (q_{k})>0$$

where $\delta (q_{k})=\mu_{t}(q_{k})-\mu_{c}(q_{k})$ for k=1,…,K. Note that, setting *q_K_* = 1, $S_{+}(q_{K})$ is the full population such that the framework also includes the possibility to perform a test in the overall population.

### 2.1 A step function model

**Figure 1.**
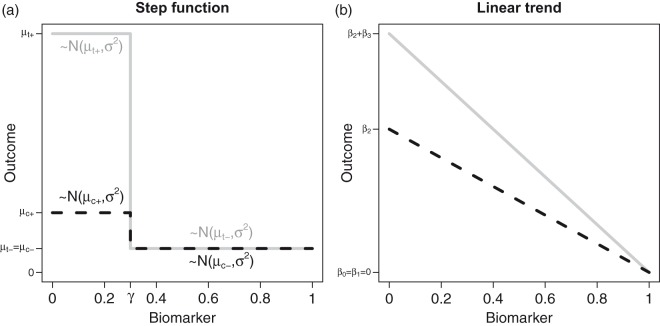
Dependence of the outcome on the biomarker value. (a)A step function dependence and (b) a linear dependence investigated in the simulation studies. Here, $\mu_{t+},\mu_{t-}$ denote the mean outcomes in the biomarker positive and negative subpopulations, respectively. The corresponding mean outcomes in the control group are denoted by $\mu_{c+}$ and $\mu_{c-}$. *β_i_*, i=0,…,3 are the regression coefficients of model (1).

A statistical model corresponding to the above analysis strategy can be written as a special case of equation (1). For a given threshold γ∈[0,1] we define

(3) $$\begin{array}{c} g(X)=\{\begin{array}{cc} 0 & \text{ if }X>\gamma \\ 1 & \text{ if }X\leq \gamma \end{array} \end{array}$$

and set $f_{1}(X)=f_{2}(X)=g(X)$ in equation (1). Then the subpopulation $S_{+}(\gamma )$ is prognostic if $|\beta_{2}|>0$ and predictive if $|\beta_{3}|>0$. Figure 1(a) shows an example where the subpopulation is predictive and prognostic, i.e. the experimental treatment has a larger effect in the subpopulation of subjects with a biomarker value smaller or equal than the cut-off *γ* as compared to the control treatment only.

### 2.2 A linear trend model

An alternative model is a linear trend model, where $f_{1}(X)=f_{2}(X)=(1-X)$ and the prognostic and predictive effects of the biomarker on the outcome *Y* are linear in the biomarker. For $|\beta_{2}|>0$ the biomarker has a prognostic effect and for $|\beta_{3}|>0$ the biomarker is predictive. See Figure 1(b) for a scenario where $\beta_{0}=\beta_{1}=0$ and the treatment effect decreases with increasing values of the biomarker *X*.

Note that under the global null hypothesis stating that all $H_{0k}$ are true, the biomarker may still have a prognostic effect. As a consequence, the marginal distribution of the outcome may no longer be normal and the variance $\sigma_{l}^{2}(q_{k})$ for l={t,c} may not be constant over the different subpopulations $S_{+}(q_{k})$. In the step function model, for example, the data in the full population follows a mixture distribution of two normal distributions where the two components correspond to the biomarker positive and negative subjects. In contrast, the subgroup $S_{+}(\gamma )$, and all subpopulations defined by thresholds smaller than *γ*, contains only biomarker positive subjects. Consequently, the variability in $S_{+}(\gamma )$ will be lower than in the full population.

## 3 Multiple hypotheses tests

Because multiple hypotheses are tested, the testing procedure needs to adjust for multiplicity to ensure strong control of the family-wise type 1 error rate (FWER) at pre-specified level *α*. A procedure controls the FWER in the strong sense if the probability that at least one true null hypothesis is rejected, is bounded by *α*, regardless of how many or which null hypotheses are holding. For the procedures given below, we investigate the FWER control under the global null hypothesis of no treatment effect in any of the subpopulations which implies weak FWER control only. However, strong FWER control follows by the closed testing principle since for all considered procedures it is easy to see that the rejection region for the test of hypotheses $H_{0}(q_{k}),k\in J$ is contained also in the rejection region of the test of $H_{0}(q_{k}),k\in J'$ for all J'⊆J⊆{1,…,K}.

### 3.1 Procedures based on multiple z- or t-tests

Assume that each hypothesis $H_{0k}$ is tested with a separate, parallel group Student’s *t*-tests based on subjects in $S_{+}(q_{k})$. Let $T(q_{k})$ denote the corresponding *t*-statistics and assume that the null hypothesis $H_{0k}$ is rejected if $T(q_{k})>c_{\alpha }(q_{k})$, where $c_{\alpha }(q_{k}),k=1,\ldots ,K$ denote critical boundaries for the tests of the subpopulations. We assume that the variance estimates used in the calculation of the *t*-statistics $T(q_{k})$ are calculated based on observations from the subpopulation $S_{+}(q_{k})$ only. Because the biomarker values of subjects *X_i_* are random (due to the sampling of subjects), in general the per-group sample sizes $n_{t}(q_{k})$ and $n_{c}(q_{k})$ in the subgroups $S_{+}(q_{k})$ will not be balanced, even if they are balanced in the full population. However, with increasing sample size, the allocation ratio in the subgroups converges to the allocation ratio in the full population.

#### 3.1.1 The Šidák test

The Šidák test applies significance levels $\alpha_{c}=1-(1-\alpha {)}^{1/K}$ and is exact if the test statistics are independent and strictly conservative if there is a positive dependence between test statistics.^11,12^ Because of the nested nature of the subgroups, the test statistics $T(q_{k})$ are positively dependent and thus the Šidák test controls the FWER in the strong sense. To apply the Šidák test, we apply the critical values $\Psi_{\mathrm{df}}^{-1}(1-\alpha_{c})$, where $\Psi_{\mathrm{df}}^{-1}$ is the quantile function of the central *t*-distribution with $\mathrm{df}=n_{t}(q_{k})+n_{c}(q_{k})-2$ degrees of freedom.

#### 3.1.2 Group sequential critical boundaries

In group sequential designs, a null hypothesis is tested repeatedly on accumulating data. Adjusted critical boundaries are applied to account for the multiple testing of the hypotheses.^8,13^ These boundaries are calculated while accounting for the correlation of the test statistics. Because the nested structure of the analysis populations at different interim analyses correspond to that of the subgroups $S_{+}(q_{k})$ defined by increasing thresholds $q_{k},k=1,\ldots ,K$, applied to a continuous biomarker, it has been proposed to use group sequential methods to derive critical boundaries for the test of nested subpopulations.^9^

Group sequential type boundaries can be derived for *z*-tests, assuming that the variance is known. For each threshold *q_k_*, we define the *z*-statistic

(4) $$Z(q_{k})=\frac{{\bar{y}}_{t}(q_{k})-{\bar{y}}_{c}(q_{k})}{\sqrt{\frac{\sigma_{t}^{2}(q_{k})}{n_{t}(q_{k})}+\frac{\sigma_{c}^{2}(q_{k})}{n_{c}(q_{k})}}},k=1,\ldots K$$

where ${\bar{y}}_{t}(q_{k})\;\text{ and }\;{\bar{y}}_{c}(q_{k})$ denote the estimated treatment and control group means of the outcomes in subpopulation $S_{+}(q_{k})$ and $\sigma_{t}^{2}(q_{k})\;\text{ and }\;\sigma_{c}^{2}(q_{k})$ the variances of the outcomes which are assumed to be known. Under the assumption of equal variances across subpopulations such that $\sigma_{t}^{2}(q_{k})=:\sigma_{t}^{2},\sigma_{c}^{2}(q_{k})=:\sigma_{c}^{2},k=1,\ldots ,K$, the correlation structure of the test statistics is the same as in group sequential designs. Then, under the null hypothesis $\delta (q_{k})=0,k=1,\ldots ,K$, the cumulative test statistics $Z(q_{k})$ follow a multivariate normal distribution with mean vector 0, variances equal to one, and covariances $\mathrm{Cov}(Z(q_{j}),Z(q_{k}))=\sqrt{I(q_{j})/I(q_{k})}$ for *q_j_* < *q_k_*, where the information $I(q_{k})$ is defined as the reciprocal of the variance of the estimated mean difference in subgroup $S_{+}(q_{k})$ such that $I(q_{k})=(\sigma_{t}^{2}/n_{t}(q_{k})+\sigma_{c}^{2}/n_{c}(q_{k}){)}^{-1}$. Assuming equal variances in the treatment and control groups, we obtain

(5) $$\mathrm{Cov}(Z(q_{j}),Z(q_{k}))=\sqrt{\frac{1/n_{t}(q_{k})+1/n_{c}(q_{k})}{1/n_{t}(q_{j})+1/n_{c}(q_{j})}}1\mathrm{mu}$$

for *q_j_* < *q_k_*, such that the covariance does not depend on the individual variances. Now, to control the level *α*, the critical boundaries $c_{\alpha }(q_{k})$ for *K* subpopulation tests have to satisfy

(6) $$1-\Phi_{0,\Sigma }(c_{\alpha }(q_{1}),\ldots ,c_{\alpha }(q_{K}))\leq \alpha$$

where $\Phi_{0,\Sigma }$ denotes the cumulative distribution function of the multivariate normal distribution with mean vector 0 and covariance matrix Σ calculated using equation (5). The level *α* condition (6) does not uniquely specify the critical value and so several families of critical boundaries have been proposed for the group sequential setting. Here we focus on Pocock type boundaries^8^ and assume that the same critical value $c_{\alpha }=c_{\alpha }(q_{k}),k=1,\ldots ,K$ is used for all subpopulation tests. Note that alternatively O’Brien Fleming type boundaries^8^ could be used, which apply larger critical levels to smaller subgroups such that these tests only reject if very large treatment effects are observed in such groups. Alongside Pocock and O’Brien Fleming boundaries, any other families of group sequential boundaries can be chosen to define the critical values.^14^

If Student’s *t*-tests to account for unknown variance instead of *z*-tests are applied at each stage, Jennison and Turnbull^8^ propose to calculate the critical values as above (based on the multivariate normal distribution) and then to transform them to the corresponding boundary of the univariate *t*-distribution with $n_{t}(q_{k})+n_{c}(q_{k})-2$ degrees of freedom. The transformed boundaries based on univariate *t*-distributions are then given by

(7) $$t_{\alpha }(q_{k})=\Psi_{n_{t}(q_{k})+n_{c}(q_{k})-2}^{-1}(\Phi_{0,1}(c_{\alpha }(q_{k})))$$

#### 3.1.3 Multiple t-tests accounting for different variances across subgroups

Due to prognostic effects of the biomarker, the variances may vary across subgroups. Then the distributional assumptions on which the group sequential approach to calculate the critical boundaries is based on, are no longer met. However, the test statistics will still asymptotically follow a multivariate normal distribution, and under the global null hypothesis $\mu_{t}(q_{k})=\mu_{c}(q_{k}),k=1,\ldots ,K$, and for thresholds $q_{j}\leq q_{k}$, the covariances are given by

(8) $$\mathrm{Cov}(Z(q_{j}),Z(q_{k}))=\frac{\sigma_{t}^{2}(q_{j})/n_{t}(q_{k})+\sigma_{c}^{2}(q_{j})/n_{c}(q_{k})}{\sqrt{\sigma_{t}^{2}(q_{j})/n_{t}(q_{j})+\sigma_{c}^{2}(q_{j})/n_{c}(q_{j})}\sqrt{\sigma_{t}^{2}(q_{k})/n_{t}(q_{k})+\sigma_{c}^{2}(q_{k})/n_{c}(q_{k})}}$$

Assuming equal variances across treatment arms within a given subgroup this simplifies to

(9) $$\mathrm{Cov}(Z(q_{j}),Z(q_{k}))=\frac{\sigma (q_{j})}{\sigma (q_{k})}\sqrt{\frac{1/n_{t}(q_{k})+1/n_{c}(q_{k})}{1/n_{t}(q_{j})+1/n_{c}(q_{j})}}$$

where $\sigma^{2}(q_{k})=\sigma_{t}^{2}(q_{k})=\sigma_{c}^{2}(q_{k}),k=1,\ldots ,K$.^6^ The covariance can be estimated by plugging the point estimates of the subgroup variances into equation (9). Then, a normal approximation of the level *α* condition is given by $1-\Phi_{0,\Sigma }(c_{\alpha },\ldots ,c_{\alpha })\leq \alpha ,$ where the covariance matrix Σ is given by equation (9). The resulting critical values are then adjusted for the finite sample case based on equation (7). Note that the proposed boundaries differ from the approach described in Placzek and Friede,^6^ where the critical boundaries are derived from a multivariate *t*-distribution approximation with a single degrees of freedom parameter. The latter is either chosen based on the smallest subgroup, leading to conservative procedures or on the total population, leading to a liberal test. In contrast, our approach is based on a multivariate normal approximation which is then adjusted for the unknown variance by quantile substitution based on univariate *t*-distributions. The degrees of freedom for each *t*-distribution are given by the size of the subgroups. While this approach is also approximate, it allows to adjust for the substantially different sample sizes across subgroups and makes the calculation of the critical boundaries computationally easier.

### 3.2 Regression models to adjust for prognostic biomarkers

An alternative approach to account for prognostic effects is a regression model for the treatment comparison. For example, adjusting for the biomarker as a covariate, we fit in each subpopulation $S_{+}(q_{k}),$ a linear regression model

Y=β0'+β1'U+β2'X+ε'

Then, for each subpopulation $S_{+}(q_{k})$, we test the null hypotheses H0k:β1'(qk)≤0 with the test statistic T(qk)=β^1'(qk)/Var(β^1'(qk)), where β^'1(qk)  and  Var(β^'1(qk)) are the standard linear model least squares estimates for the parameter and its variance.

The correlation structure of the test statistics can be approximated based on the group sequential approach by estimating the information for subgroup $S_{+}(q_{k})$ by I(qk)=(Var(β^'(qk)))-1. Then critical boundaries $c_{\alpha }(q_{k})$ can be calculated using the multivariate normal distribution with covariance $\mathrm{Cov}(Z(q_{j}),Z(q_{k}))=\sqrt{I(q_{j})/I(q_{k})}$ for *j* < *k*.^8^ To adjust for the unknown variance, we recalculate the boundaries as in equation (7) but based on a univariate *t*-distribution with $n_{t}(q_{k})+n_{c}(q_{k})-3$ degrees of freedom.

Similar as for the group sequential *t*-test, the calculation of the critical boundaries relies on the assumption that the variance of the residuals is the same in all subpopulations. Thereby this approach extends the group sequential approach to the setting of prognostic biomarkers. While the assumption of a common variance across subpopulations hold if the fitted regression model is correct, the residual variances may vary across subpopulations if the model is misspecified.

### 3.3 Inverse normal combination tests

A multiple testing procedure for nested subpopulations can also be constructed using combination tests.^10,13,15,16^ To this end we split the population into disjoint subsets

$$S_{+}(q_{k-1},q_{k})=\{i:q_{k-1}<x_{i}\leq q_{k}\},k=1,\ldots ,K$$

where $q_{0}=0$. Then, in each subset $S_{+}(q_{k-1},q_{k})$ a Student’s *t*-test and corresponding *p*-value $p(q_{k-1},q_{k})=1-\Psi_{\mathrm{df}}(T(q_{k-1},q_{k}))$ is calculated, $T(q_{k-1},q_{k})$ denoting the test statistics calculated using patients in $S_{+}(q_{k-1},q_{k})$ only. These *p*-values are combined with a combination function, as, for example, the weighted inverse normal combination function^10^ to obtain the test statistics

(10) $$C(q_{k})=\sum_{m=1}^{k}\sqrt{\frac{w_{m}}{\sum_{j=1}^{k}w_{j}}}\Phi^{-1}\left(1-p(q_{m-1},q_{m})\right)$$

where $w_{k}=[1/n_{c}(q_{k-1},q_{k})+1/n_{t}(q_{k-1},q_{k}){]}^{-1}$ and $n_{l}(q_{k-1},q_{k}),l\in \{t,c\}$ denote the number of subjects in the treatment and control group in subset $S_{+}(q_{k-1},q_{k})$. The individual *p*-values are independent under the global null hypothesis. Furthermore, assuming the data in each subset are normally distributed, the *p*-values are uniformly distributed on [0,1]. It follows that the test statistics $C(q_{k})$ are multivariate normally distributed with a correlation structure of a group sequential test with information levels $I_{k}(q_{k})=\sum_{i=1}^{k}\frac{w_{i}}{\sum_{j=1}^{k}w_{j}},\;k=1,\ldots ,K$.^10^ Therefore, the corresponding group sequential critical boundaries $c_{\alpha }(q_{k})$ as derived in equation (6) can be applied to obtain a test with FWER *α*. Note that the weights *w_k_* weigh the contribution of the subsets $S_{+}(q_{k-1},q_{k})$ accounting for the different subpopulation sample sizes in the treatment and control group.

## 4 Properties of the multiple testing procedures

To investigate the operating characteristics of the procedures introduced in the previous section, a simulation study was performed. For simplicity, we assume the biomarker to be uniformly distributed on [0,1] and investigate hypotheses tests for *K* = 2, 4 and 8 thresholds. The thresholds are equally spaced such that $q_{k}=k/K,k=1,\ldots K$. Especially, *q_K_* = 1 and also the full population is tested. For the boundaries based on the group sequential approaches, equal critical values $c_{\alpha }(q_{k})=c_{\alpha },k=1,\ldots ,K$ were computed. The nominal FWER was set to α=0.025. For each scenario, $5\cdot 10^{5}$ simulation runs were performed. Group sequential critical boundaries were calculated using the R-package *gsDesign*.^17^

We considered six testing procedures: Šidák adjusted *t*-tests, *t*-tests based on critical values $c_{\alpha }$ as in equation (6), further on denoted by “z-test”, the corresponding *t*-tests based on the adjusted critical values $t_{\alpha }(q_{k})$ as in equation (7) denoted by “*t*-test”, the *t*-test accounting for different variances using equation (9) denoted by “adjusted *t*-test”, the test based on the regression model and the test based on the inverse normal method.

The data were generated based on the model given in equation (1) with per group sample sizes of *n* = 80. Simulation results for the FWER for *n* = 160 and 320 can be found in the Supplementary material.

We considered two scenarios. First, the step function model defined in equation (3) (see Figure 1(a)) with parameters γ=0.2,0.5,0.8 was considered. The FWER was evaluated in settings where there is no treatment effect in any subgroup but possibly a prognostic effect, i.e. for subjects with biomarker smaller than *γ*, the expected outcome is $\mu_{t+}=\mu_{c+}=\Delta$ while for the remaining subjects the expected outcome is $\mu_{t-}=\mu_{c-}=0$ and Δ varies between 0 and 3. To evaluate the power of the procedures, we set $\mu_{c+}=\mu_{c-}=\mu_{t-}=0$ (no prognostic effect) and assumed that the treatment had only an effect in subjects with biomarker X≤γ. There the effect sizes varied between 0 and 1 standard deviations. We report simulation results under the alternative hypothesis for a sample size of *n* = 80 per group.

The second scenario considered is the linear trend model (see Figure 1(b)), where the prognostic and predictive effects of the biomarker on the outcome *Y* are linear. We considered settings where $\beta_{0}=\beta_{1}=0$ and, for the simulations under the null hypothesis of no treatment effect, in addition that $\beta_{3}=0$. However, we allowed for a prognostic effect (i.e. $\beta_{2}\geq 0$). For the simulations under the alternative hypothesis, we set $\beta_{0}=\beta_{1}=\beta_{2}=0$ and varied *β*_3_ between 0 and 1, such that the treatment effect decreases with the value of the biomarker *X*.

In both scenarios, the variance of the noise term *ε* in equation (1) was set to 1.

### 4.1 Family-wise Type 1 error rate

**Figure 2.**
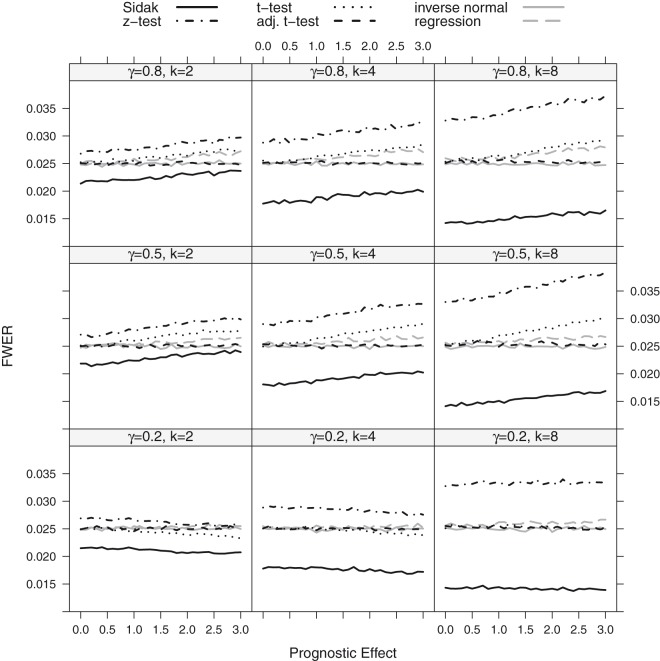
FWER as a function of the prognostic effect assuming a step function dependence for a sample size of *n* = 80. The number of thresholds was set to *K* = 2, 4, 8 with true cut-off γ=0.2,0.5,0.8. The black lines show the results for the Šidák (solid), the *z*-test (dot-dashed), the *t*-test (dotted) and the adjusted *t*-test (dashed) while the grey lines represent the regression procedure (dashed) and the inverse normal test (solid).

**Figure 3.**
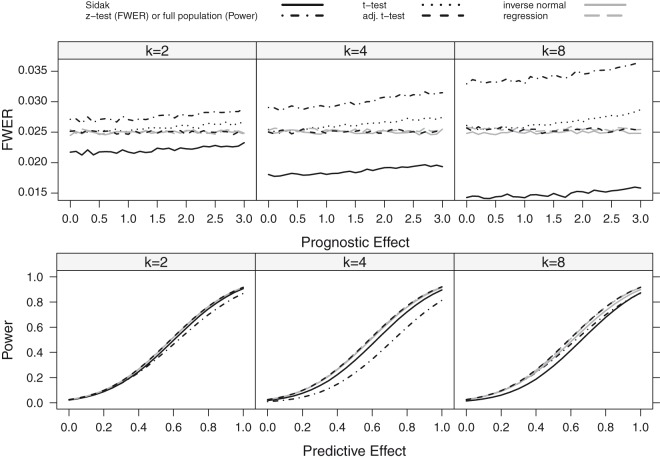
FWER and Power as a function of the prognostic (FWER-Plot) or predictive (Power-Plot) effect assuming a linear dependence for a sample size of *n* = 80. The number of thresholds was set to *K* = 2, 4, 8. The black lines show the results for the Šidák (solid), the *z*-test (FWER-Plot) or full population test (Power-Plot) (dot-dashed), the *t*-test (dotted) and the adjusted *t*-test (dashed) while the grey lines represent the regression procedure (dashed) and the inverse normal test (solid).

The FWER for the considered procedures is shown in Figures 2 and 3 for the step function model and the linear trend model. If there is no prognostic effect, all considered methods control the FWER, with the exception of the *z*-test (which is only based on the normal approximation) which has an inflated error rate for the scenarios with small to moderate sample sizes.

If the biomarker has, however, a prognostic effect, also the group sequential boundaries adjusted with *t*-quantiles (*t*-test) can become liberal. The amount of inflation depends on the size of the prognostic effect, the number of thresholds considered (the more thresholds, the larger the inflation) and the value of the true cut-off point *γ*. The observed inflation results from the effect of the prognostic effect on the variance of the outcome in the different subgroups. For larger prognostic effects, the variance of the outcomes in the different subgroups vary and this has an impact on the correlation structure between test statistics such that the assumptions underlying the computation of the critical boundaries based on a group sequential test are no longer satisfied. This leads in several settings to an inflation of the FWER when using group sequential boundaries. Note that (with the exception of the z-test with low or moderate sample sizes) substantial inflations of the FWER are only observed for prognostic effects larger than a standard deviation.

The regression procedure has a somewhat lower FWER for the step function model but remains anti-conservative because of the model misspecification. In the linear trend model, it controls the level well.

Across all scenarios, the *t*-test accounting for different subpopulation variances (adjusted t-test), the inverse normal combination test and the Sidák test control the FWER. However, the latter is strictly conservative, especially for a larger number of thresholds.

### 4.2 Power

**Figure 4.**
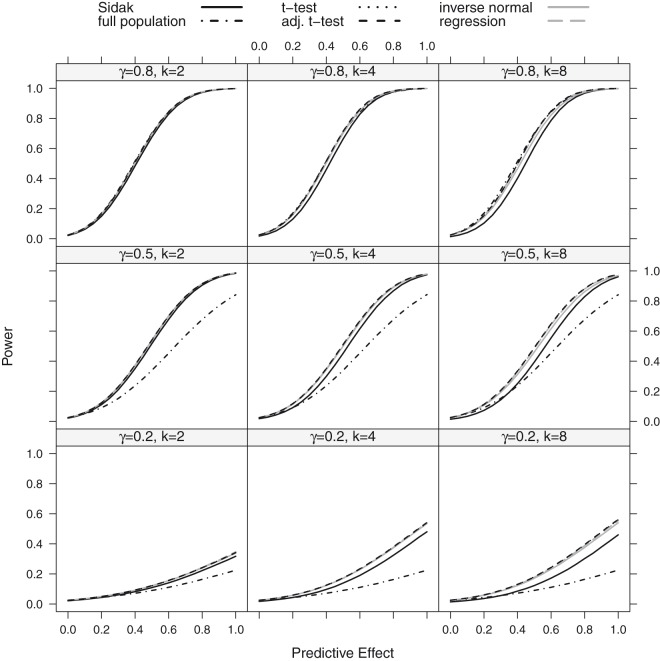
Power as a function of the predictive effect assuming a step function dependence for a sample size of *n* = 80: the number of thresholds was set to *K* = 2, 4, 8 with true cut-off γ=0.2,0.5,0.8. The black lines show the results for the Šidák (solid), the full population test (dot-dashed), the *t*-test (dotted) and the adjusted *t*-test (dashed) while the grey lines represent the regression procedure (dashed) and the inverse normal test (solid).

We report the power of the procedures, defined as the probability to reject at least one of the *K* hypotheses. We did not consider the *z*-test in these simulations, as it did not sufficiently control the FWER for the considered sample size of *n* = 80. Instead, we also report the power of a single *t*-test in the full population, for comparison. The power for the step function and the linear trend model is shown in Figures 3 and 4. Under both scenarios, the approaches based on group sequential *t*-tests (*t*-test, adjusted *t*-test), the regression approach and the inverse normal combination test show similar power and the lines in the plot are partly indistinguishable. For *K* = 8 thresholds, the inverse normal combination test has a somewhat lower power compared to the group sequential *t*-test and the regression method because of a loss of degrees of freedom due to the split in disjoint subsets. Over all scenarios, the Šidák test shows the lowest power as it does not make full use of the correlation structure between the test statistics. If the size of the subpopulation is small (γ=0.2) or moderate (γ=0.5), the power for the single *t*-test in the full population is much lower than the power for the multiple testing procedures that test for a treatment effect in several subgroups. If the subpopulation is large (γ=0.8), the single full population test has a similar power as compared to the multiple testing methods and may exceed their power if the number of considered subgroups *K* becomes too large and the loss in power due to the multiplicity correction outweighs a potential increase in efficiency by testing in a subpopulation with a larger treatment effect.

### 4.3 Model misspecifications

In the above simulations, the structure of the tested subgroups (where all patients with a biomarker value below a certain threshold are included) is in agreement with the considered scenarios, where the prognostic and predictive effects decrease monotonically with the biomarker (see Figure 1). To assess the robustness of the procedure, we investigated FWER and power if this assumption does not hold. As above, we assumed a step function model and a linear trend model but with monotonically increasing prognostic and predictive effects such that $f_{1}(X)=f_{2}(X)=1-g(X)$ or $f_{1}(X)=f_{2}(X)=X$, respectively. In addition, we performed simulations where the largest prognostic and predictive effects are observed for intermediate values of the biomarker. These misspecifications have no impact on the FWER and the simulated FWERs are similar to those of the correctly specified model. The adjusted *t*-test, the Šidák test and the inverse normal combination test control the FWER in all scenarios, the Šidák test being conservative. The sequential regression test controls the FWER under a linear trend dependence. The *z*-test and the *t*-test show an inflated FWER for increasing prognostic effects. However, misspecification of the subgroups can have a substantial negative impact on the power. The largest power has the test for the full population only. Among the considered multiple testing procedures, the mutliple *t*-test, the regression and the inverse normal combination test procedure have similar power values, and the Šidák test showed somewhat smaller power values. For detailed simulation results, see the supplemental material.

## 5 Example: clinical trials in depression with a predictive biomarker

Depression is a common and disabling disease for which a number of pharmacological and psychosocial treatment options are available. However, no single treatment is universally effective and the response to treatment is slow and hard to predict. Therefore, many patients with depression undergo multiple treatments before achieving remission.^1,2^ One problem is the heterogeneity of the disease which has motivated the investigation of biomarkers to predict the treatment outcome. As outcome measures, in such studies often the decrease in a score describing the severity of the disease is used. Examples of commonly used instruments include the Montgomery-Asberg Depression Rating Scale (MADRS), the Hamilton Rating Scale for Depression (HRSD) or the Beck-Depressions-Inventar II Score (BDI-II).

Luty et al.^18^ compared in a randomized controlled trial interpersonal psychotherapy (IPT) and cognitive-behavioural therapy (CT) for major depression. A total of 177 patients were randomly allocated to the two treatment groups. As primary outcome variable, the percentage improvement in MADRS score from baseline to the end of a 16-week treatment phase was investigated. No statistically significant difference between IPT and CT was found for the full population. In a secondary analysis, however, investigators found that severely depressed patients responded significantly better to CT than to IPT, suggesting baseline severity as a predictor for response. To categorize severe depression, they used a fixed threshold for the baseline MADRS score. No correction for multiplicity was performed for the subgroup test.

Similarly, Lemmens et al.^19^ compared IPT and CT in a randomized controlled trial also concluding that there is no statistically significant difference between the two treatments. The main outcome measure was the decrease in BDI-II score from baseline to seven months. Also 182 patients were randomized in three groups, 75 to IPT, 76 to CT and 31 patients were randomized to a waiting list control condition. Although no statistically significant difference between the two active treatment arms was observed, both treatments were superior to the waiting list group. In a re-analysis of the data based on the IPT and CT groups only, Huibers et al.^20^ investigated several baseline scores (describing the severity of the disease), as, e.g., the Inventory of Interpersonal Problems Score (IIP), the Beck Hopeless Scale (BHS), the Brief Symptom Inventory (BSI) or quality of life scores as potential predictors for treatment outcome. Using a variable selection approach based on linear regression models with interaction terms, they found that, for example, the BSI Cognitive Problems score or the IIP self-sacrificing score may be moderators of treatment outcome.

To illustrate the statistical methods discussed in this manuscript, we used the trial data which is available at DRYAD ^21^ comparing the IPT group (*n* = 75) to the CT group (*n* = 76). As outcome measure, we used the difference between BDI-II score from baseline to seven months. The reduction in BDI-II Score in the full population was 14.59 (±15.14) for the IPT group as compared to 14.39 (±15.97) in the CP group. For illustration, we assume that it was planned to investigate whether the baseline total IIP score is a predictive biomarker of the BDI-II reduction. In the planning phase of the trial, the thresholds for the subpopulation analyses were pre-specified at the theoretical 25%, 50%, 75% and 100% percentiles (the actual sample sizes will differ due to sampling variation). The observed baseline IIP Score ranged from 16 to 164, the thresholds are set to $q_{1}=64,q_{2}=89,q_{3}=107$ and $q_{4}=164$ which correspond to the 25%, 50%, 75% and 100% percentiles, respectively. Table 1 shows the mean values and standard deviation as well as test statistics and the corresponding critical boundaries for the discussed methods. A larger test statistics indicates a larger reduction in the BDI-II score from baseline to seven months of the IPT as compared to the CT. Although the cumulative test statistics show a trend, that patients with smaller IIP Score values benefit more from IPT as compared to CT, no statistically significant differences were observed in the subgroups.

**Table 1.** Mean (SD) of the outcome, test statistics and critical boundaries of the Šidák, *z*-test, *t*-test and the corrected *t*-test as well as for the regression method and the inverse normal test separately for the four nested subgroups calculated from the example data set.

	25%	50%	75%	100%
IPT: reduction BDI-II	14.94 (11.68)	15.81 (9.42)	15.16 (7.08)	14.59 (15.14)
CT: reduction BDI-II	9.86 (7.72)	12.56 (10.17)	13.91 (10.66)	14.39 (15.97)
Cumulative *t*-test statistics for				
Šidák, *z*-test, *t*-test, adjusted *t*-test	1.63	1.39	0.63	0.10
Boundary *t*-test	2.46	2.41	2.39	2.38
Boundary adjusted *t*-test	2.49	2.43	2.41	2.40
Boundary Šidák	2.62	2.56	2.54	2.53
Cumulative regression test statistics	1.65	1.56	0.75	0.02
Boundary regression test	2.47	2.41	2.40	2.39
Inverse normal test statistics	1.59	1.89	1.31	1.03
Boundary *z*-test and inverse normal	2.35	2.35	2.35	2.35

## 6 Multiple *t*-test for general subgroups

Consider a general set of subsets $B_{1},\ldots ,B_{K}\subseteq \mathbb{R}$ and let $\mu_{t}(q_{k})=E(Y|U=1,X\in B_{k}),\mu_{c}(q_{k})=E(Y|U=0,X\in B_{k})$ denote the means and $\sigma_{t}^{2}(B_{k}),\sigma_{c}^{2}(B_{k})$ the respective variances of the outcome *Y* in the subpopulations defined by *B_k_*. As above, we test the *K* null hypotheses

$$H_{0k}:\delta (q_{k})\leq 0\text{ against }H_{1k}:\delta (q_{k})>0$$

where $\delta (q_{k})=\mu_{t}(q_{k})-\mu_{c}(q_{k})$ for k=1,…,K. Let $Z(B_{k})$ denote the corresponding *z*-test statistics for the observations in the subgroups $S_{+}(B_{k})=\{i:X_{i}\in B_{k}\}$ in analogy to equation (4). Then the covariance between the test statistics of subgroups $S_{+}(B_{k}),S_{+}(B_{k'})$ is given by

(11) $$\mathrm{Cov}(Z(B_{k}),Z(B_{k'}))=\frac{\frac{\sigma_{t}^{2}(B_{k}\cap B_{k'})n_{t}(B_{k}\cap B_{k'})}{n_{t}(B_{k})n_{t}(B_{k'})}+\frac{\sigma_{c}^{2}(B_{k}\cap B_{k'})n_{c}(B_{k}\cap B_{k'})}{n_{c}(B_{k})n_{c}(B_{k'})}}{\sqrt{\frac{\sigma_{t}^{2}(B_{k})}{n_{t}(B_{k})}+\frac{\sigma_{c}^{2}(B_{k})}{n_{c}(B_{k})}}\sqrt{\frac{\sigma_{t}^{2}(B_{k'})}{n_{t}(B_{k'})}+\frac{\sigma_{c}^{2}(B_{k'})}{n_{c}(B_{k'})}}}$$

The normal approximation of the level *α* condition is given by $1-\Phi_{0,\Sigma }(c_{\alpha },\ldots ,c_{\alpha })\leq \alpha ,$ where the covariance matrix Σ is given by equation (11) where the variances and covariances are replaced by sample estimates. As above, the quantile substitution method can be used to adjust the critical values for the appropriate degrees of freedom. Furthermore, instead of choosing equal critical values *c* for all subgroups, a vector of individual critical values *c_k_* satisfying the level-*α* condition can be chosen.

An application of this more general procedure are subgroups defined by the tail-oriented construction of the STEPP method,^5^ which has been proposed in settings where there is uncertainty if very low or very large values of the biomarker are predictive for a large treatment effect. Here, first a left-to-right cumulation of patient values is performed where subgroups are defined by all subjects with biomarker values below a set of thresholds (as defined in the above sections) and then a right-to-left cumulation is performed where subgroups are defined by all subjects with biomarker values above the set of thresholds. Given *K* thresholds, this procedure defines 2K-1 subsets (assuming the largest threshold is ∞ such that the test of the full population is included).

## 7 Discussion

We investigated methods for nested subpopulation tests, where the subpopulations are defined by thresholds of a continuous biomarker.

Our results show that special care has to be taken when using critical boundaries from group sequential designs, as has been proposed previously. If there are prognostic effects that are not adequately adjusted for, the standard critical boundaries from group sequential designs will not control the FWER in general. However, a substantial inflation of the FWER occurs for large prognostic effects only. Correcting for the least favorable correlation structure using the Šidák test controls the FWER. However, it can become very conservative if a larger number of subgroups are tested when it also leads to a loss in power. The inverse normal combination test controls the FWER but has slightly smaller power for a larger number of thresholds due to a loss in degrees of freedom.

Furthermore, the power calculations show that if the subpopulation, where the treatment effect is positive, is large, testing the null hypothesis for the full population only has similar power as compared to the multiple testing methods testing for a treatment effect in multiple subgroups. However, in settings where the subgroup where the treatment effect is positive, is smaller, the multiple tests have a larger power to reject at least one null hypothesis than the test for the full population only. The findings on the power imply that in these settings, the sample size required to achieve a certain power is lower for the multiple testing procedure than for the single test in the full population (accounting for the diluted treatment effect in the latter). The sample size yielding a certain power for the multiple testing procedures cannot be given explicitly, but can be obtained through numerical approximation or simulation techniques (see e.g. Placzek and Friede^6^). For example, assuming a true cut-off in the step function model of γ=0.5, a sample size of 54 per group is needed to detect a treatment effect of one standard deviation in the biomarker positive subgroup assuming no effect in the biomarker negative subgroup with power 0.9 using the adjusted *t*-test with *K* = 4 equally spaced thresholds and α=0.025. If, however, γ=0.2, a sample size of 180 per group is needed under the above assumptions. Note that a true cut-off of γ=0.5 (or 0.2 respectively) corresponds under the given assumptions to an effect size of 0.5 (0.2) standard deviations in the full population. With a single *t*-test in the full population, therefore 86 (527) patients per group are needed to achieve a power of 0.9. Similar examples can be found in Placzek and Friede.^6^

Note that for the sample size calculation the thresholds must be chosen in the planning phase of a trial because the critical boundaries for the multiple *t*-test depend on the number of thresholds *K* as well as the size of the subgroups. If the subgroups are defined by absolute thresholds (rather than quantiles), the sample size calculation will be based on expected subgroup sizes since the actual subgroup sizes are random. In this case, at the final analysis the critical values need to be updated based on the actual subgroup sizes. Alternatively one may choose the thresholds based on quantiles of the continuous biomarker such that the subgroup sizes are fixed. This, however, results in data-dependent absolute thresholds.

In this manuscript, we focused on single-step multiple testing procedures. Using the closed testing principle, these can be improved by a sequentially rejective test. While this has no impact on the probability to reject at least one null hypothesis, it will increase the power to demonstrate a statistically significant treatment effect in several subgroups. Furthermore, for all the considered testing, multiplicity adjusted *p*-values can be defined by determining for each hypothesis the smallest significance level *α*, for which the test rejects the respective hypothesis.

The observed FWER inflation for group sequential tests of hypotheses for nested subpopulations has also implications for classical group sequential designs. A corresponding type 1 error rate inflation can occur also in group sequential tests of a single hypothesis if there is a time trend in the outcome variable. The calendar time then has a similar impact as the prognostic biomarker in the subpopulation tests and the classical group sequential test may have an inflated type 1 error rate.

An alternative approach to test for a treatment effect in nested subpopulations that has not been explored in this manuscript is to fit a single linear model including the factors treatment, as well as indicator functions of the disjoint sets $S_{+}(q_{k-1},q_{k})$ and their interaction with the factor treatment. The treatment effect in each subgroup $S_{+}(q_{k})$ can then be estimated as a suitable contrast and simultaneous hypothesis tests can be derived by multiple contrast tests which have been implemented in the *multcomp* package in R.^22^

## Supplemental Material

Supplemental material for Robustness of testing procedures for confirmatory subpopulation analyses based on a continuous biomarker Click here for additional data file. Supplemental Material for Robustness of testing procedures for confirmatory subpopulation analyses based on a continuous biomarker by Alexandra Christine Graf, Gernot Wassmer, Tim Friede, Roland Gerard Gera and Martin Posch in Statistical Methods in Medical Research
