# Supplementary material for: Robustness of testing procedures for confirmatory subpopulation analyses based on a continuous biomarker
Source: Stat Methods Med Res. 2018 Jun 11;28(6):1879–92. doi: 10.1177/0962280218777538 (PMC6566459; doi:10.1177/0962280218777538)
Supplement: Supplemental material for Robustness of testing procedures for confirmatory subpopulation analyses based on a continuous biomarker [file Supplemental_Material.pdf]

# Supplemental Material to: Robustness of testing procedures for confirmatory subpopulation analysis based on a continuous biomarker

Alexandra Christine Graf[a], Gernot Wassmer[a], Tim Friede[b], Roland Gera[b] and Martin Posch[a,†]

[a] Center for Medical Statistics, Informatics and Intelligent Systems, Medical University of Vienna, Spitalgasse 23, 1090 Vienna, Austria

[b] Department of Medical Statistics, University Medical Center Goettingen Humboldtallee 32, 37073 Goettingen, Germany

In addition to the simulations discussed in the manuscript for a sample size of $n = 80$ we performed simulations to evaluate the Family-wise Type 1 Error Rate (FWER) with two more sample sizes ($n = 160$ and $n = 320$ per group) for the step function dependence and linear dependence as shown in Figure 1 of the manuscript. The results of these simulations are shown in Section 1.

Furthermore, we simulated the FWER and Power for the scenarios shown in Figure S1 for a sample size of $n = 80$. Scenario (A) and (B) give a setting with a reverse dependence as compared to Scenarios (A) and (B) from Figure 1 showing a prognostic and/or predictive effect for patients with larger biomarker values. Scenario (C) in Figure S1 shows a setting with a prognostic and/or predictive effect for patients with intermediate biomarker values around 0.5. The results of this simulations will be shown in Section 2.

Similar to the simulations shown in the main manuscript with $n = 80$ (Figures 2 - 4), we assumed the biomarker to be uniformly distributed on $[0, 1]$ and investigate hypotheses tests for $K = 2, 4$ and 8 thresholds. The thresholds are equally spaced such that $q_k = k/K, k = 1, \dots K$. For the boundaries based on the group sequential approaches, equal critical values $c_\alpha(q_k) = c_\alpha, k = 1, ..., K$ were computed. The nominal FWER was set to $\alpha = 0.025$. For each scenario $5 \cdot 10^5$ simulation runs were performed.

For the FWER simulations we considered six testing procedures: Šidák tests, t-tests based on critical values $c_\alpha$, further denoted as "z-test", the corresponding t-tests based on the adjusted critical values $t_\alpha(q_k)$ denoted by "t-test", the t-test accounting for different variances denoted by "adjusted t-test", the test based on the regression model denoted by "regression" and the test based on the inverse normal method denoted by "inverse normal". For the power simulations we report the power of a t-test of the full population instead of results of the z-test which does not control the FWER.

†corresponding author: e-mail: martin.posch@meduniwien.ac.at

# 1 Sample Size $n = 160$, $320$ per Group

The data was generated based on the model (1) of the manuscript and with per group sample sizes of $n = 160$ and 320. We considered two scenarios. First, the step function model as shown in Figure 1 (A) of the main manuscript was investigated where $f_1(X) = f_2(X) = g(X)$ in model (1). The parameter $\gamma$ was set to $0.2, 0.5, 0.8$. For the investigation of the FWER we considered settings where there is no treatment effect in any subgroup but possibly prognostic effect, i.e., for subjects with biomarker smaller than $\gamma$ the expected outcome is $\mu_{t+} = \mu_{c+} = \Delta$ while for the remaining subjects the expected outcome is $\mu_{t-} = \mu_{c-} = 0$ and $\Delta$ varies between 0 and 3.

The second scenario was the linear trend model as shown in Figure 1 (B) with $f_1(X) = f_2(X) = 1 - X$ in model (1). The prognostic and predictive effects of the biomarker on the outcome $Y$ are linear. We considered settings where $\beta_0 = \beta_1 = 0$ and, for the simulations under the null hypothesis of no treatment effect, in addition to that $\beta_3 = 0$. However, we allowed for a prognostic effect (i.e. $\beta_2 \geq 0$) which varied between 0 and 3, such that the treatment effect decreases with the value of the biomarker $X$. In both scenarios the variance of the noise term $\epsilon$ in (1) was set to 1.

Figures S2 - S4 show the simulation results for the FWER assuming step function dependence and a per-group sample size of $n = 160$ (Figure S2) and $n = 320$ (Figure S3). Figure S4 shows the simulation results for the FWER assuming a linear dependence for per-group sample sizes of $n = 160$ and $n = 320$.

# 2 Model Misspecifications

The data was generated for a per group sample size of $n = 80$. In all scenarios the variance was set to 1. We considered three different scenarios.

First, the step function model as shown in Figure S1 (A) was investigated where $f_1(X) = f_2(X) = 1 - g(X)$ in model (1). The biomarker positive subgroup includes all patients with a biomarker value larger than $\gamma_m$. The parameter $\gamma_m$ was set to $0.2, 0.5$ and $0.8$. Note that if e.g. $\gamma_m = 0.8$ then the biomarker positive subgroup includes 20% of the full population. For the investigation of the FWER we considered settings where there is no treatment effect in any subgroup but possibly a prognostic effect, i.e., for subjects with biomarker larger than $\gamma_m$ the expected outcome is $\mu_{t+} = \mu_{c+} = \Delta$ while for the remaining subjects the expected outcome is $\mu_{t-} = \mu_{c-} = 0$ and $\Delta$ varies between 0 and 3. To evaluate the power of the procedures, we set $\mu_{c+} = \mu_{c-} = \mu_{t-} = 0$ (no prognostic

effect) and assumed that the treatment had only an effect in subjects with biomarker $X > \gamma_m$ where the effect sizes varied between 0 and 1 standard deviations. Results for the FWER are shown in Figure S5 and for the power in Figure S6.

The second scenario was the linear trend model as shown in Figure S1 (B) with $f_1(X) = f_2(X) = X$. We considered settings where $\beta_0 = \beta_1 = 0$ and, for the simulations under the null hypothesis of no treatment effect, $\beta_3 = 0$. However, we allowed for a prognostic effect (i.e. $\beta_2 \geq 0$) which varied between 0 and 3, such that the treatment effect increases with the value of the biomarker $X$. For the simulations under the alternative hypothesis we set $\beta_0 = \beta_1 = \beta_2 = 0$ and varied $\beta_3$ between 0 and 1, such that the treatment effect increases with the value of the biomarker $X$. Results for the FWER are shown in Figure S7 and for the power in Figure S8.

In the third scenario as shown in Figure S1 (C) we assumed that patients in the biomarker positive subgroup have biomarker values between $0.5 - \lambda$ and $0.5 + \lambda$. Under the null hypothesis we assumed the outcome to be normally distributed with mean $\mu_{t+} = \mu_{c+} = \Delta$ in the biomarker positive subgroup and $\mu_{t-} = \mu_{c-} = 0$ in the biomarker negative subgroup with the prognostic effect $\Delta$ varying from 0 to 3. The parameter $\lambda$ was set to 0.1, 0.25, and 0.4 meaning that the intermediate 20%, 50% or 80% are in the biomarker positive subgroup. To evaluate the power of the procedures we set $\mu_{c+} = \mu_{c-} = \mu_{t-} = 0$ (no prognostic effect) and assumed that the treatment had only an effect in subjects with biomarker $0.5 - \lambda \leq X \leq 0.5 + \lambda$. There the effect sizes varied between 0 and 1 standard deviations. Results for the FWER are shown in Figure S9 and for the power in Figure S10.

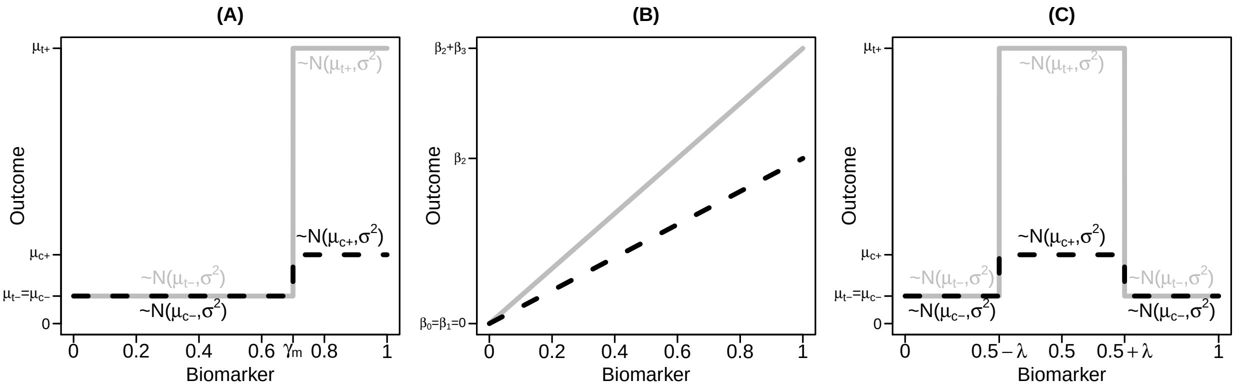


Figure S1: Dependence of the outcome on the biomarker value. Figure (A) shows a step function dependence and Figure (B) a linear dependence investigated in the simulation studies. Figure (C) shows a step function dependence with the prognostic and/or predictive effect for patients with a biomarker value around 0.5.

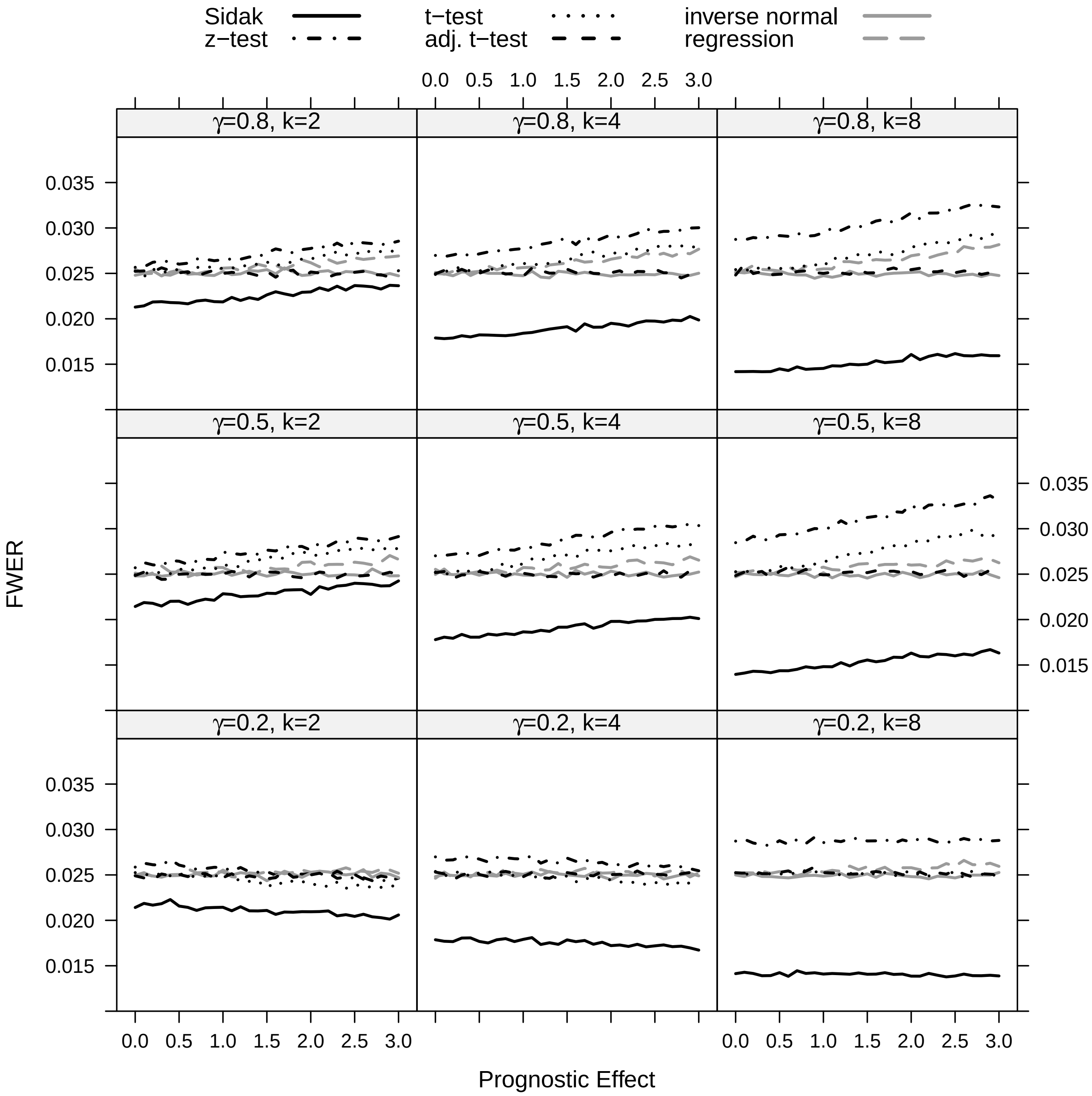


Figure S2: FWER simulation results as a function of the prognostic effect assuming a step function dependence as in Figure 1 (A) for a samples size of $n = 160$: the number of thresholds was set to $K = 2, 4, 8$ with true cut-off $\gamma = 0.2, 0.5, 0.8$. The black lines show the results for the Šidák (solid), the z-test (dot-dashed), the t-test (dotted) and the adjusted t-test (dashed) while the grey lines represent the regression procedure (dashed) and the inverse normal test (solid).

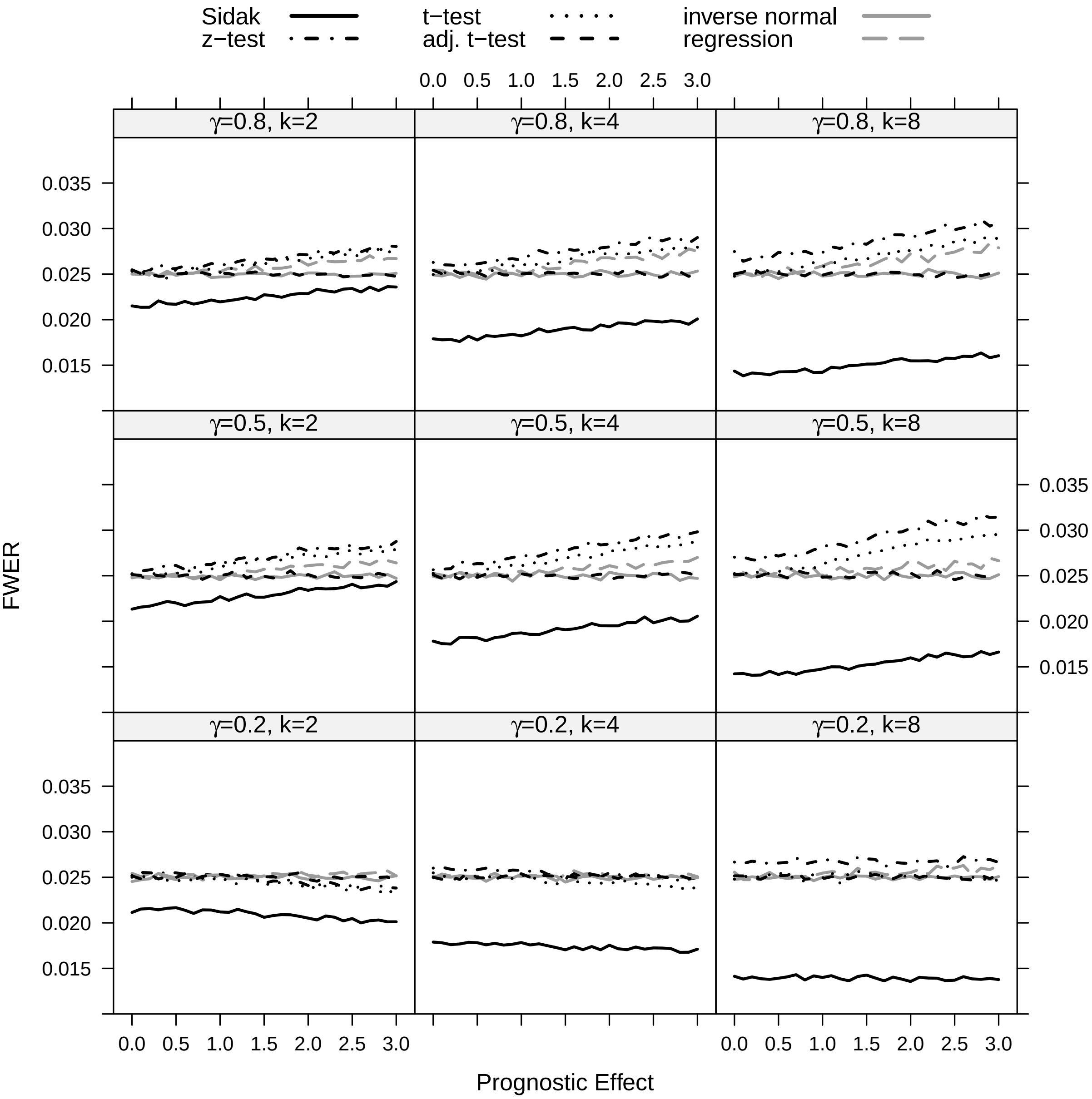


Figure S3: FWER as a function of the prognostic effect assuming a step function dependence as in Figure 1 (A) for a sample size of $n = 320$. The number of thresholds was set to $K = 2, 4, 8$ with true cut-off $\gamma = 0.2, 0.5, 0.8$. The black lines show the results for the Šidák (solid), the z-test (dot-dashed), the t-test (dotted) and the adjusted t-test (dashed) while the grey lines represent the regression procedure (dashed) and the inverse normal test (solid).

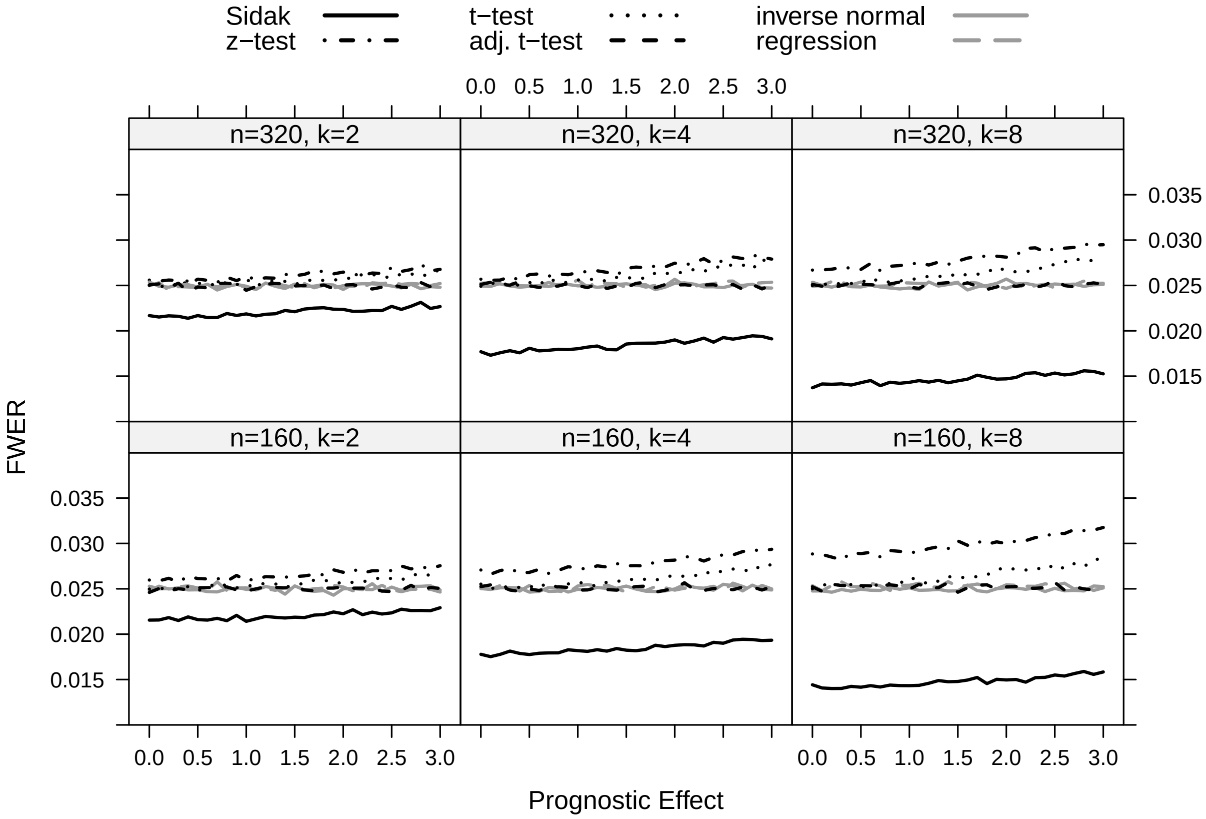


Figure S4: FWER as a function of the prognostic effect assuming a linear dependence as in Figure 1 (B) for a samples size of $n = 160$ and $n = 320$. The number of thresholds was set to $K = 2, 4, 8$. The black lines show the results for the Šidák (solid), the z-test (dot-dashed), the t-test (dotted) and the adjusted t-test (dashed) while the grey lines represent the regression procedure (dashed) and the inverse normal test (solid).

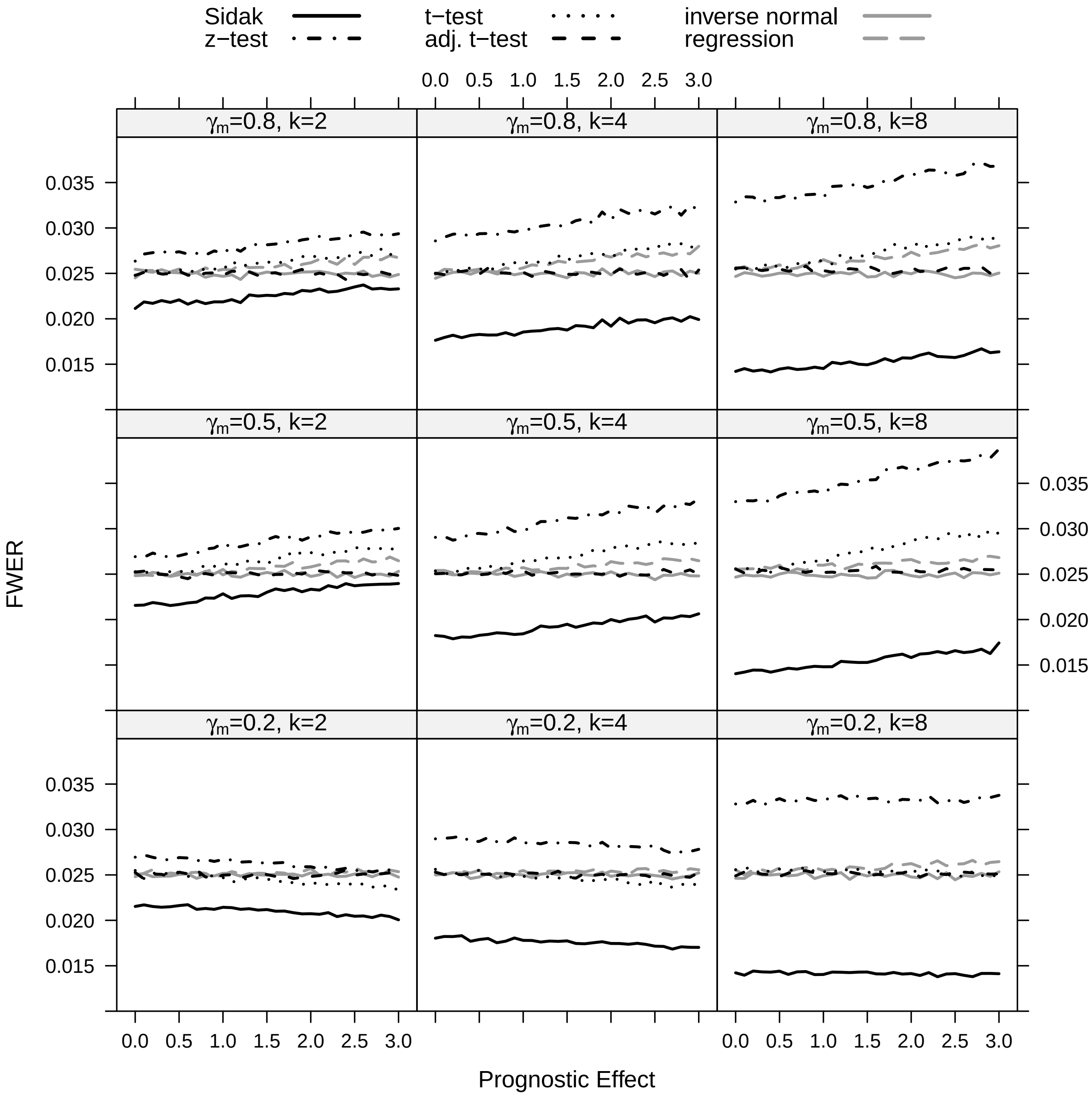


Figure S5: FWER simulation results as a function of the prognostic effect assuming a step function dependence as in Figure S1 (A) for a sample size of $n = 80$: the number of thresholds was set to $K = 2, 4, 8$ with true cut-off $\gamma_m = 0.2, 0.5, 0.8$. The black lines show the results for the Šidák (solid), the z-test (dot-dashed), the t-test (dotted) and the adjusted t-test (dashed) while the grey lines represent the regression procedure (dashed) and the inverse normal test (solid).

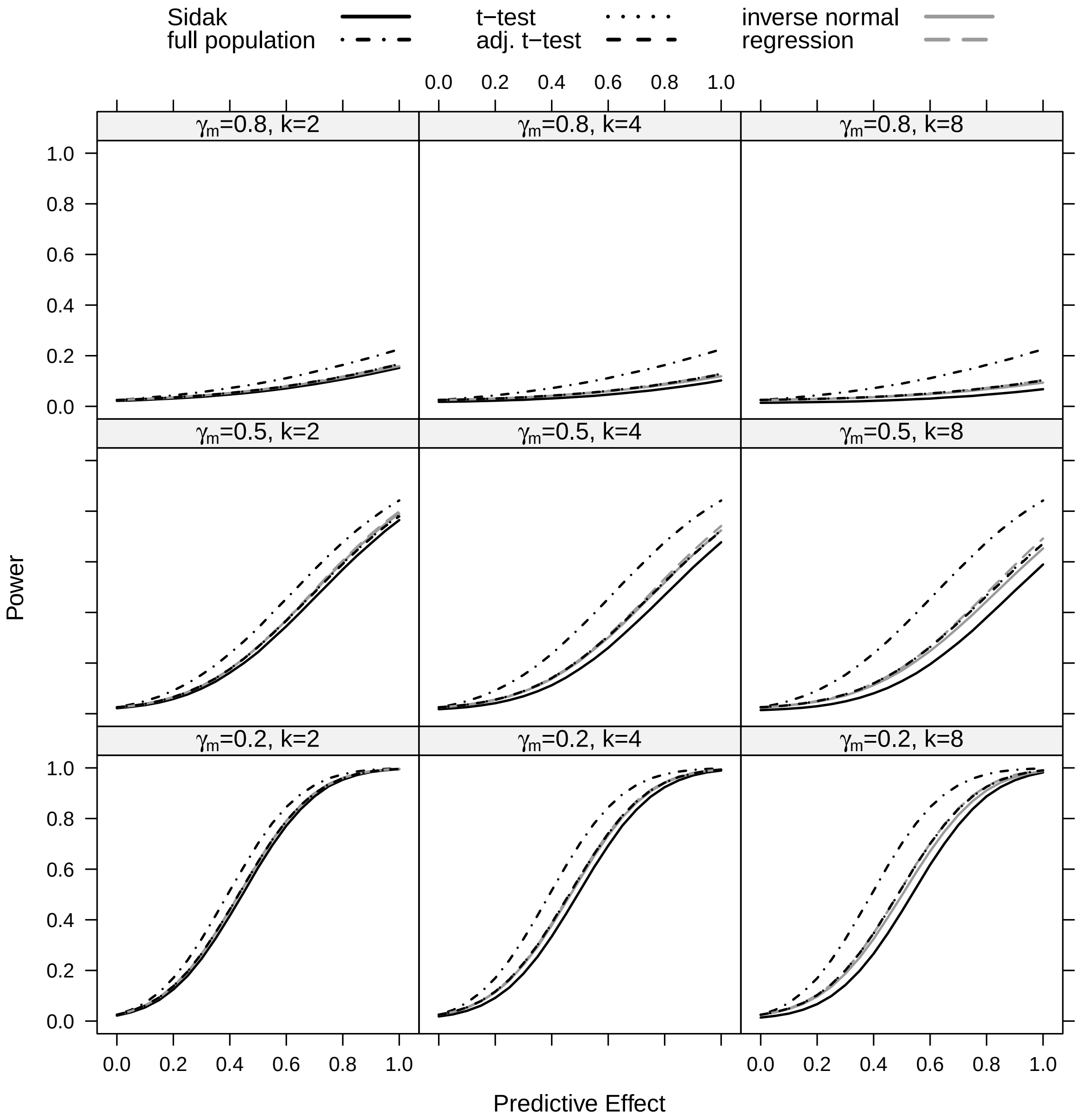


Figure S6: Power simulation results as a function of the predictive effect assuming a step function dependence as in Figure S1 (A) for a sample size of $n = 80$: the number of thresholds was set to $K = 2, 4, 8$ with true cut-off $\gamma_m = 0.2, 0.5, 0.8$. The black lines show the results for the Šidák (solid), the full population test (dot-dashed), the t-test (dotted) and the adjusted t-test (dashed) while the grey lines represen the regression procedure (dashed) and the inverse normal test (solid).

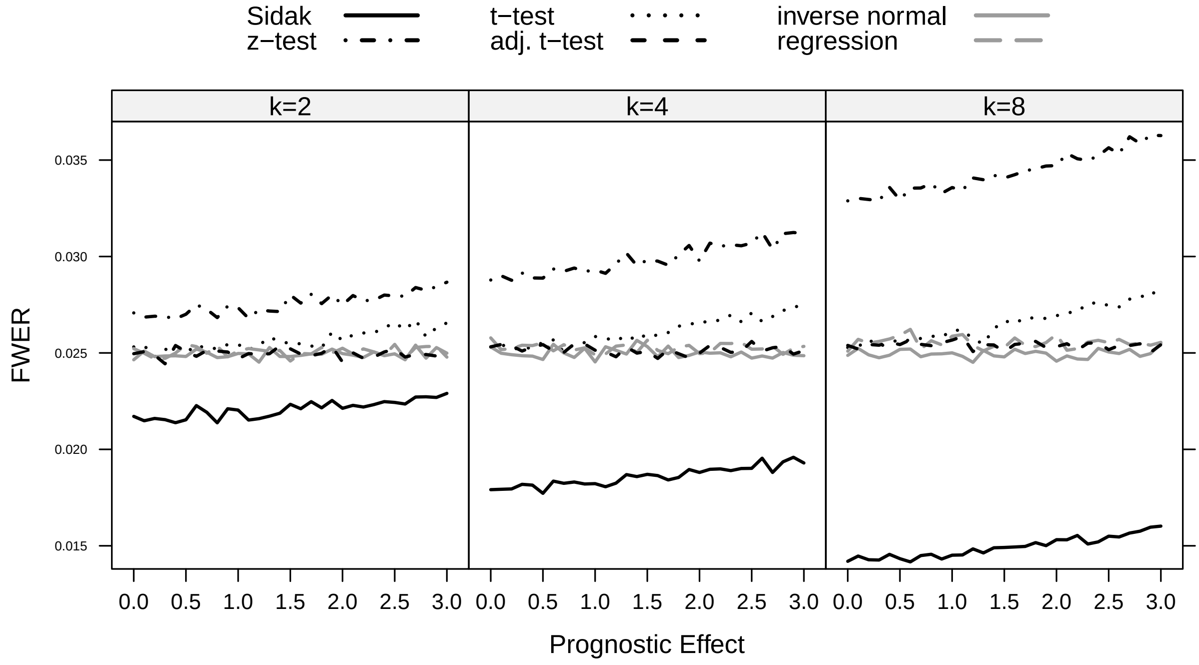


Figure S7: FWER as a function of the prognostic effect assuming a linear dependence as in Figure S1 (B) for a sample size of $n = 80$. The number of thresholds was set to $K = 2, 4, 8$. The black lines show the results for the Šidák (solid), the z-test (dot-dashed), the t-test (dotted) and the adjusted t-test (dashed) while the grey lines represent the regression procedure (dashed) and the inverse normal test (solid).

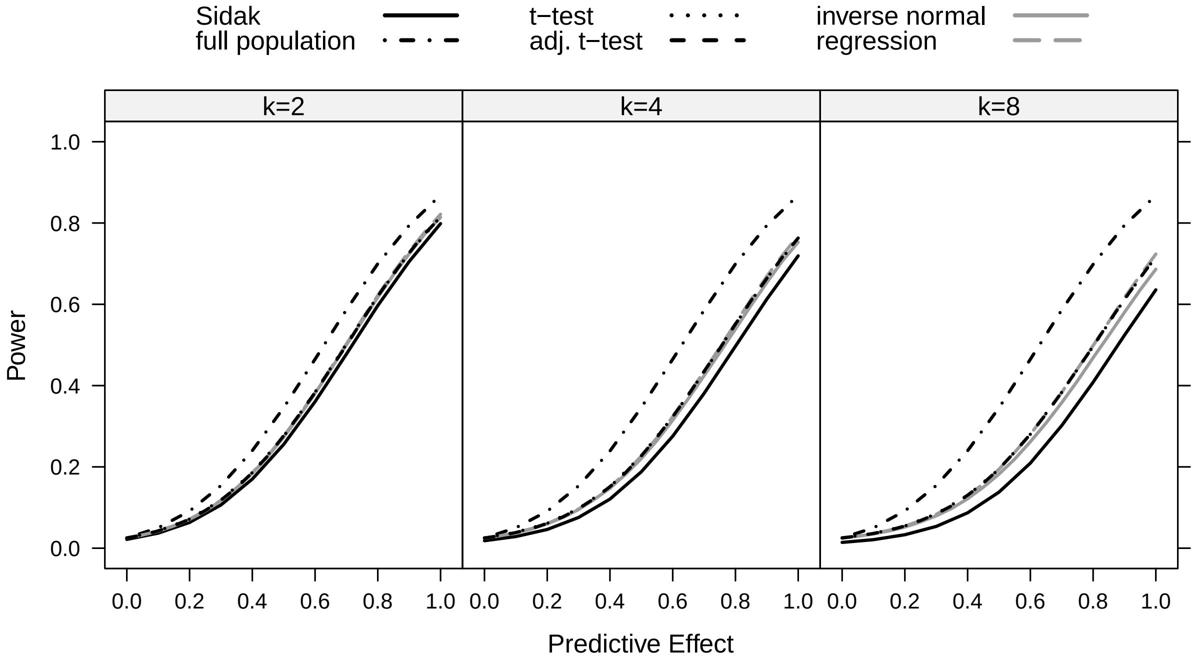


Figure S8: Power simulation results as a function of the predictive effect assuming a linear dependence as in Figure S1 (B) for a sample size of $n = 80$. The number of thresholds was set to $K = 2, 4, 8$. The black lines show the results for the Šidák (solid), the full population test (dot-dashed), the t-test (dotted) and the adjusted t-test (dashed) while the grey lines represent the regression procedure (dashed) and the inverse normal test (solid).

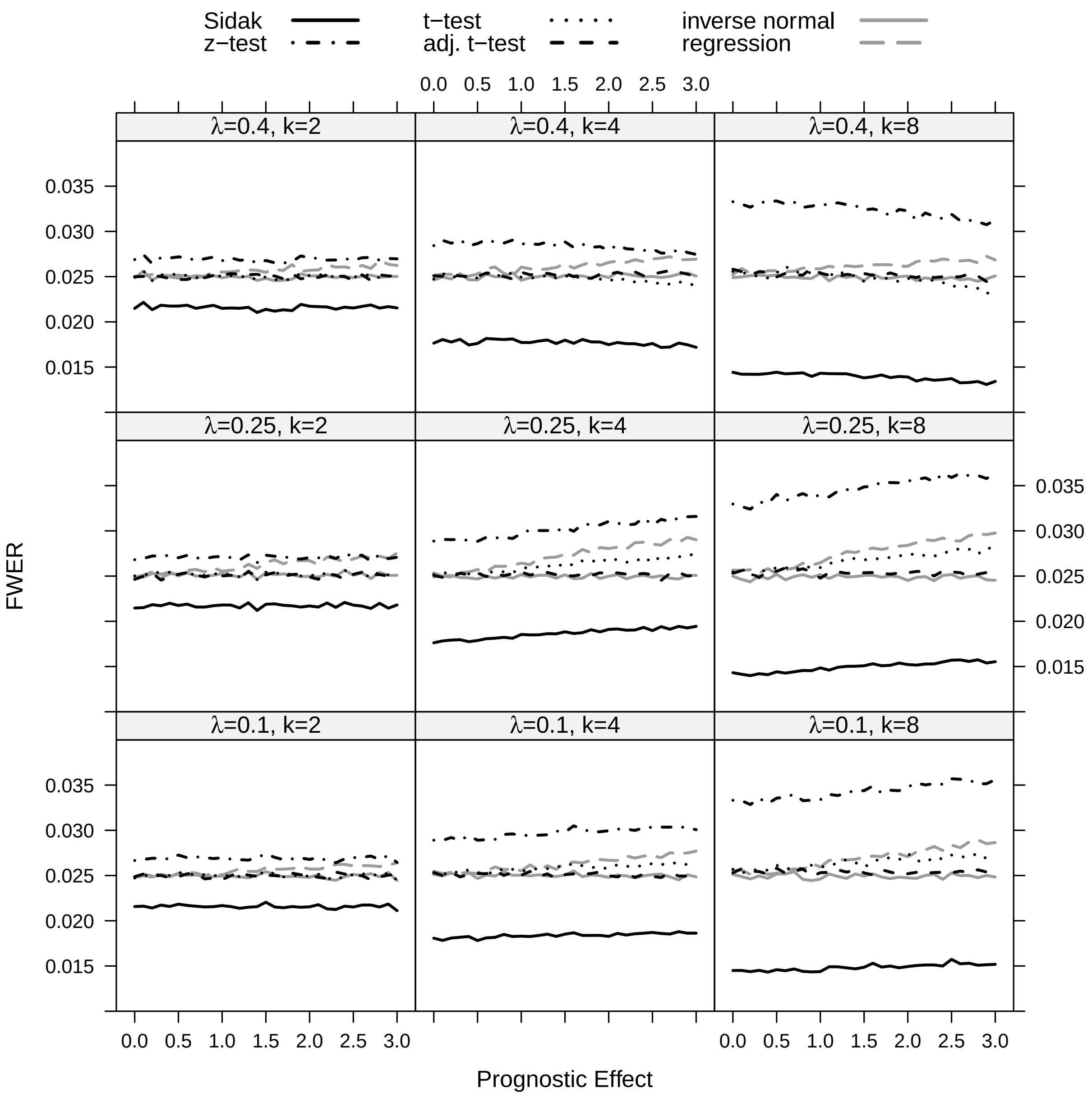


Figure S9: FWER simulation results as a function of the prognostic effect assuming a step function dependence as in Figure S1 (C) for a sample size of $n = 80$: the number of thresholds was set to $K = 2, 4, 8$ and the parameter $\lambda = 0.1, 0.25, 0.4$. The black lines show the results for the Šidák (solid), the z-test (dot-dashed), the t-test (dotted) and the adjusted t-test (dashed) while the grey lines represent the regression procedure (dashed) and the inverse normal test (solid).

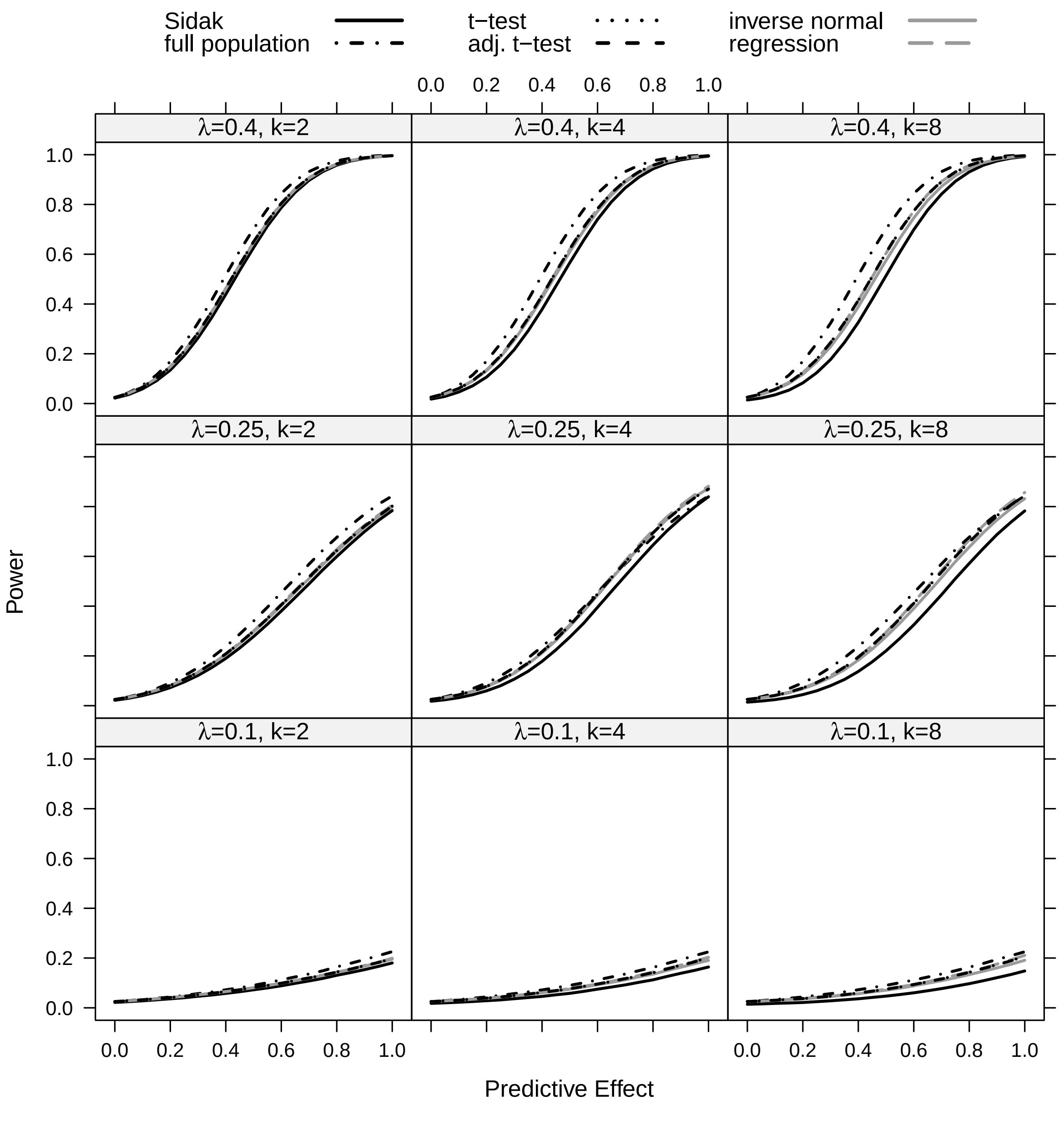


Figure S10: Power simulation results as a function of the predictive effect assuming a step function dependence as in Figure S1 (C) for a sample size of $n = 80$: the number of thresholds was set to $K = 2, 4, 8$ and the parameter $\lambda = 0.1, 0.25, 0.4$. The black lines show the results for the Šidák (solid), the full population test (dot-dashed), the t-test (dotted) and the adjusted t-test (dashed) while the grey lines represent the regression procedure (dashed) and the inverse normal test (solid).
